# Long-term follow-up of residual symptoms in patients treated for stress-related exhaustion

**DOI:** 10.1186/s40359-020-0395-8

**Published:** 2020-03-19

**Authors:** Kristina Glise, Lilian Wiegner, Ingibjörg H. Jonsdottir

**Affiliations:** 1 Institute of Stress Medicine, Region Västra Götaland, Carl Skottsbergs gata 22B, SE-413 19 Gothenburg, Sweden; 2 School of Public Health and Community Medicine, Institute of Medicine, Sahlgrenska Academy at the University of Gothenburg, Gothenburg, Sweden

**Keywords:** Exhaustion, Stress, Symptoms, Recovery

## Abstract

**Background:**

Many patients with stress-related exhaustion seem to struggle with long-term recovery. The primary aim of this study was to explore residual symptoms and perceived recovery in patients previously treated for stress-related exhaustion, 7 years after seeking care.

**Methods:**

A total of 217 former patients (74% women) previously treated for exhaustion disorder were asked to participate in follow-ups 2, 3, 5, and 7 years post treatment. Symptoms of depression, and anxiety were measured with questionnaires. Remaining symptoms of extreme fatigue, sleep disturbances, problems with concentration, problems with memory and reduced stress tolerance, were rated with single item questions. A subgroup of patients (*n* = 163) participated in a clinical assessment to confirm residual stress-related exhaustion not caused by other diseases.

**Results:**

Almost half of the patients previously treated for stress-related exhaustion perceive fatigue 7 years after initially seeking care, and as many as 73% reported decreased stress tolerance. The clinical assessment confirmed that a third of the patients were clinically judged as still suffering from stress-related exhaustion. Male and female patients showed similar patterns regarding residual symptoms.

**Conclusions:**

One third of patients with exhaustion disorder are clinically judged to have exhaustion, 7 years after seeking care. Further studies are needed to elucidate the reason for such a long-term recovery and ultimately to identify methods for prevention.

## Background

The past decade has seen several descriptions of patients seeking care for severe symptoms of exhaustion and cognitive impairment due to both work-related and private-related stress exposure [1–3]. Core symptoms include extreme fatigue, sleep disturbances, cognitive impairment and reduced stress tolerance, often in combination with symptoms of depression and anxiety and somatic symptoms such as headache and stomach problems [1, 4]. These clinical attributes go beyond the original definition of the psychological term “burnout”, which assume the symptoms to be solely work-related. Thus, burnout was defined as a negative emotional reaction to work, resulting in exhaustion and social withdrawal, depersonalization and reduced personal accomplishment [5, 6]. As early as the mid-1980s it was argued that a distinction should be made between burnout as a work-related stress syndrome and burnout as a clinical mental disability [7, 8]. Symptoms of exhaustion and burnout are closely related [1, 9] but the most utilized burnout tool, the Maslach Burnout Inventory (MBI), does not seem to be suitable as a diagnostic tool for patients [10]. In the Netherlands, clinical burnout has been suggested as a diagnosis, using diagnostic criteria such as neurasthenia and adding the component that the problem should be work-related [11]. In Sweden, the increased number of patients seeking care for stress-related exhaustion called for an action of improving diagnostics in cases of stress-related exhaustion/clinical burnout. This resulted in the development of the criteria-based diagnosis exhaustion disorder (ED). The criteria was proposed by an expert group working for the National Board of Health and Welfare in Sweden in 2003 and they have then gradually been implemented in clinical practice in Sweden [2]. The criteria were assigned the code F43.8A of the International Classification of Diseases and Related Health Problems (ICD-10) (Table 1).

**Table 1:** Diagnostic criteria for Exhaustion Disorder according to the National Board of Health and Welfare (2003)

A	Physical and mental symptoms of exhaustion with minimum two weeks duration. The symptoms have developed in response to one or more identifiable stressors which have been present for at least 6 months.
B	Markedly reduced mental energy, which is manifested by reduced initiative, lack of endurance, or increase of time needed for recovery after mental efforts.
C	At least four of the following symptoms have been present most of the day, nearly every day, during the same 2-week period:
*1*	*Persistent complaints of impaired memory.*
*2*	*Markedly reduced capacity to tolerate demands or to work under time pressure.*
*3*	*Emotional instability or irritability.*
*4*	*Insomnia or hypersomnia.*
*5*	*Persistent complaints of physical weakness or fatigue.*
*6*	*Physical symptoms such as muscular pain, chest pain, palpitations, gastrointestinal problems, vertigo or increased sensitivity to sounds.*
D	The symptoms cause clinically significant distress or impairment in social, occupational or other important areas of functioning.
E	The symptoms are not due to the direct physiological effects of a substance (e.g. a drug of abuse, a medication) or a general medical condition (e.g. hypothyroidism, diabetes, infectious disease).
F	If criteria for major depressive disorder, dysthymic disorder or generalized anxiety disorder are met, exhaustion disorder is set a co-morbid condition.

The severe mental burden of illness and cognitive impairments described by patients with exhaustion, together with indications that brain function might be affected, raises the question of how long recovery is needed following stress-related exhaustion [2]. We have previously shown that after 18 months of treatment, one third of patients still report symptoms of burnout; this was not related to sex or age [1]. Longer follow-up periods of similar groups indicate that recovery can be long-lasting for some but not all patients suffering from burnout/exhaustion. Hätinen and co-workers reported that despite a year of rehabilitation and an additional 6 months of follow-up, as many as 37% of patients did not show any recovery regarding symptoms of burnout [12]. Stenlund and co-workers showed that around two thirds of clinical burnout patients reported mental fatigue and physical fatigue 3 years after seeking care [13]. Thus, is seems that a subgroup of ED patients experiences long-lasting functional impairments related to fatigue. To our knowledge no follow-up exceeding 3 years have been conducted and thus the overall aim of the present study was to explore residual symptoms and clinical exhaustion in patients previously diagnosed with ED, 7 years after seeking care.

## Methods

### Aim

The primary aim of this study was to explore perceived recovery, and residual symptoms including fatigue, depression and anxiety among previous ED patients 7 years after seeking care for their exhaustion. The secondary aim was to explore how many patients were still considered clinically exhausted 7 years after seeking care. The tertiary aim was to explore any differences between female and male patients regarding residual symptoms following ED.

### Participants

The patients included in this longitudinal follow-up study were former patients at a specialist clinic in Gothenburg, Sweden. Baseline characteristics measured when they initially sought care are shown in Table 2. A post-treatment register was set up for all patients who fulfilled the diagnostic criteria for ED and took part in the 12–18 months of treatment at the clinic, conducted sometimes between 2004 and 2012. The treatment has been previously described in detail [1, 4]*.* Briefly, after an extensive diagnostic procedure by a physician all patients were offered treatment with similar components but adapted to their individual needs during a treatment period lasting for approximately 18 months. The patients visited a physician every 4–6 weeks with successively longer duration between visits. When needed the patients received an individual consultation by a psychologist and psychotherapy was offered for patients in need of therapy. Recommendations regarding lifestyle factors, including regular physical activity and sleep was offered to all patients and these factors were repeatedly discussed at all visits at the clinic. Many patients received more in-depth consultation from a physiotherapist regarding graded physical activity as an important part of the treatment. Some of the patients with severe sleep disturbances were offered cognitive behavioural group therapy for insomnia at the end of rehabilitation period. Psychoeducation and/or stress reduction group programme were offered to all patients after the first consultation. Employers, work colleagues, and relatives were also invited to attend a short lecture regarding stress-related mental disorders. Antidepressant medication was offered patients with co-morbid depression and/or anxiety when needed according to clinical judgement. Finally, communication with the Social Insurance Office and the employer was facilitated, and about half of the patients participated in special meetings regarding the earliest possible return to work.

**Table 2:** Baseline characteristics of female and male patients with stress-related Exhaustion Disorder (ED), measured when they initially sought care (*N* = 217)

	Total (*n* = 217)	Women (*n* = 161)	Men (*n* = 56)
Age Mean (SD)	44 (9.2)	44 (9.2)	42 (9.2)
Percent patients married/co-living N (%)	160 (74)	120 (75)	40 (71)
Percent patients with higher Education^a^ N (%)	151 (70)	113 (70)	38 (68)
Symptom of Burnout baseline (> 4.4 SMBQ) N (%)	193 (90)	143 (89)	50 (91)

*SMBQ* Shirom Melamed Burnout Questionnaire

^a^Higher education is defined as ≥1 year of college education

**Fig. 1:**
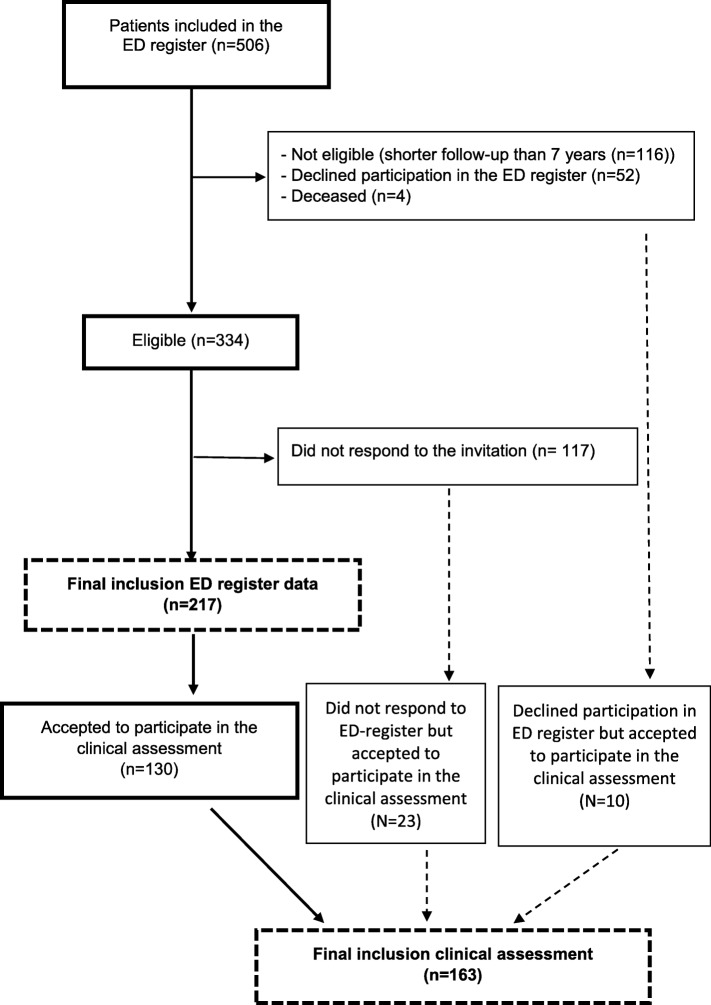
Flow chart of the total of *N* = 506 patients included in the exhaustion disorder (ED) patient register, were 334 patients had passed 7 years follow-up. A final number of 217 (65%) responded to the 7 years follow-up questionnaires, 161 women (74%) and 56 men (26%). Furthermore, a population of 163 patients accepted to participate in a clinical assessment. Sixty-percent of this population (*n* = 130) had also responded to the 7 years follow-up, The remaining patients participating in the clinical assessment either did not respond to the register follow-up but accepted the participation in the clinical assessment (*n* = 23) or declined participation in the register follow-up but accept to participate in the clinical follow-up (*n* = 10)

All patients who fulfilled the diagnostic criteria for ED when entering the program at the clinic, were asked to participate in follow-up measures. Inclusion criteria for this study was that the patients should have passed the time point of 7 years follow-up. Of the 506 patients included in the register, 116 did not fulfil the criteria of 7 years follow-up, and additional five patients were not included due to ill-health, unknown address, or disinclination to participate in further studies (see flow chart, Fig. 1). Thus, register data from 334 patients were eligible to be used in the analysis of follow-up data. Of these 334 patients, 217 (65%) responded to the 7-year follow-up and returned the questionnaires: 161 women (74%) and 56 men (26%), with a mean age of 44 years (SD 9.2) at baseline and 51 years (SD 9.2) at the 7-year follow-up.

The study was approved by the regional ethical review board in Gothenburg, Sweden (2015-10-16, ref.: 668–15). All potential participants were sent information about the study along with an informed consent form, and only those who provided their signed informed consent were included.

### Study procedures

Questionnaires including information about baseline data such as age, civil status, education and employment, sick-leave, and symptoms of depression, anxiety, and burnout were given to the patients to fill in at the clinic during their ordinary visits (baseline, 3, 6, 12, and 18 months) and during the post-treatment period (2, 3, 5, and 7 years) the questionnaires were posted to the patients. The rationale of analysing 7 years follow-up was that our clinical experience confirms that many patients report long-lasting symptoms, often several years after finishing treatment. To ensure that the symptoms plausible could be judged to be residual, data from 2, 3, and 5 years were also included. Each symptom was defined to be residual if the patient reported the symptom on at least one previous follow-up (2, 3, or 5 years) additionally to reporting the symptom at 7 years follow-up.

### Clinical assessment of exhaustion

A clinical assessment was conducted to further assess residual stress-related exhaustion. Thus, former patients who had passed the 7-year follow-up (range: 7–12 years after initially seeking care) were invited to visit the clinic for a clinical assessment. A total of 163 patients (77% women) accepted the invitation and thus were clinically assessed (se Fig. 1). The criteria used by the physicians to judge residual exhaustion were either that 1) the patient fulfilled the full diagnostic criteria for ED, or 2) the patient scored > 4.4 on the Shirom-Melamed Burnout questionnaire (SMBQ) and confirmed to have residual exhaustion according to the clinical assessment. The rationale for the second definition was that many patients do not entirely fulfil the ED criteria due to absence of single symptom but according to the clinical assessment they are clearly defined by the physician too suffer from residual symptoms due to their former ED. Other diagnoses that could plausibly explain the fatigue were also assessed. Except for 14 patients, the same two physicians (experienced general practitioner (GP) and an experienced physician with several specialities (including GP and occupational medicine) conducted the clinical judgement of the patients both at baseline and at follow-up.

### Measurements

Information regarding demographics such as age, sex, education, employment, and sick-leave was collected at baseline; that is, when the patients first visited the clinic. At the post-treatment follow-ups, the patients were asked whether they perceived symptoms related to their previous exhaustion, with yes/no response alternatives. The symptoms extreme fatigue, sleep disturbances, problems with concentration and problems with memory were chosen because they are included in the diagnostic criteria and reduced stress tolerance was included since this is a common clinical manifestation. All items were categorical (yes/no); that is, the measurement was of whether or not the patient perceived this to be a residual symptom following the exhaustion. An open question asking about other residual symptoms was also included, and the patients were asked to state which symptom(s).

The Shirom-Melamed Burnout Questionnaire (SMBQ) was used to measure symptoms of burnout/exhaustion both at baseline and at all follow-ups [14]. Originally the scale consisted of 22 items and four subscales: burnout, cognitive weariness, listlessness, and tension. Following Rasch analysis of the scale, the tension subscale was removed, leaving a reliability-tested 18-item version that was used in this study [12]. Responses are recorded on a 7-point Likert scale from 1 (“almost never”) to 7 (“almost always”), and hence the total mean score can range from 1 to 7. We have previously shown that the SMBQ can be used to follow symptoms of exhaustion over time in patients with ED [1]. This instrument measures exhaustion irrespective of the causal attribution; work-related or non-work-related. Mean scores were calculated for the total SMBQ burnout score. In the present study, a total mean score of ≥4.4 was used as the cut-off for burnout [15].

The widely used Hospital Anxiety and Depression Scale (HADS) was chosen to assess self-reported symptoms of depression and anxiety. It was originally developed for non-psychiatric clinics to detect states of depression and anxiety [16]. The scale consists of 14 statements concerning feelings during the past week, seven for each of the two subscales. Four response alternatives (scored 0–3) indicating degree or frequency are available for each statement. A sum score above 10 was used in this study to define symptoms of depression and anxiety, respectively [16]. HADS has satisfactory factor structure and internal consistency, as well as acceptable discriminant and concurrent validity [17]. It has also been shown to be sensitive in reflecting changes over time in response to different interventions [18].

### Statistics

Drop-out analysis was performed using Student’s t-test (except for age, which was not normally distributed, and so the Mann-Whitney U-test was used instead) and Pearson’s chi-square test when appropriate. Pearson’s chi-square two-tailed tests were performed to analyse possible differences between men and women regarding residual symptoms (extreme fatigue, sleep disturbances, problems with concentration, problems with memory, reduced stress tolerance) and symptoms of anxiety and depression (> 10 on HADS). The level of significance was set at *p* < 0.05. Version 22 of IBM SPSS Statistics for Windows was used for all statistical analyses.

## Results

### Comparisons with non-responders

When patients responding to the 7-year follow-up (*n* = 217) were compared to non-responders eligible to enter the study (*n* = 117), there were no significant differences regarding the percentage of women/men who participated (responders 74%/26% vs. non-responders 65%/35%; *p* = 0.076), severity of burnout at baseline (90% of responders vs. 94% of non-responders scoring above the clinical SMBQ cut-off when initially seeking care; *p* = 0.197), or severity of burnout at the end of treatment (18-months follow-up; 31% of responders vs. 36% of non-responders scoring above clinical cut-off; *p* = 0.426). The study group were significantly older (44 years; SD 9.2) compared to the non-responders (41 years; SD 8.8) at baseline (*p* = 0.003). The group agreeing to the clinical assessment did not differ from the group replying to the questionnaires regarding age, sex, educational level, or severity of burnout at baseline (data not shown).

### Sick-leave data

The patients were asked to report if they were on sick-leave (100, 75, 50, 25%, or 0%) or receiving disability pension 7 years after initially seeking care for exhaustion. The vast majority were not on sick-leave (87%; *n* = 188). Seven patients (3%) reported 100% sick-leave, 12 patients (6%) part-time sick-leave, and eight patients (4%) reported disability pension. The patients reporting sick-leave at the 7-year follow-up were asked to state which diagnosis was registered as the cause of sick-leave; all 19 patients reported diagnoses related to stress-related mental health problems (depression, anxiety or exhaustion disorder).

### Perceived recovery and residual symptoms 7 years after seeking care for stress-related exhaustion

The patients were asked to report their current health status in relation to their previous exhaustion. Only 16% (*n* = 35) of the patients reported that they were fully recovered, though a majority reported that they were much better (*n* = 125; 59%) or better (*n* = 44; 21%). The remaining 4% (*n* = 9) reported that their health was unchanged or had worsened. As shown in Table 3, the most common residual symptom was reduced stress tolerance, reported by as many as 73% (*n* = 158) of the patients. Moreover, 46% of the patients reported extreme fatigue and 43% reported problems with memory. Many patients reported more than one residual symptom. There were 21 responses to the question about other symptoms; the most commonly reported symptoms here were loss of energy (*n* = 4), increased noise sensitivity (*n* = 4), decreased stress tolerance (*n* = 3), headache/migraine (*n* = 3), and tinnitus/dizziness (*n* = 3). No difference was seen between men and women.

**Table 3:** Percentage of female and male patients reporting residual symptoms related to exhaustion 7 years after seeking care for stress-related exhaustion. Each symptom was defined to be residual if the same symptom was reported at least one timepoint during 2, 3- and 5-years follow-up and at 7 years follow-up

Symptom (N) (Women/Men)	Total N (%)	Females N (%)	% males N (%)	*p* value
Extreme fatigue (*N* = 217) (161/56)	99 (46)	76 (47)	23 (41)	.427
Sleep disturbances (*N* = 217) (161/56)	77 (36)	58 (36)	19 (34)	.778
Problems with concentration (*N* = 217) (161/56)	77 (36)	59 (37)	18 (32)	.544
Problems with memory (*N* = 217) (161/56)	90 (42)	73 (45)	17 (30)	.050
Reduced stress tolerance (*N* = 217) (161/56)	158 (73)	122 (76)	36 (64)	.096

All symptoms are measured with single item (Yes/No)

### Symptoms of depression and anxiety

Symptoms of depression according to HADS were rarely reported by the patients at the 18 months follow-up (post-treatment assessment) (6% among female patients and 7% among male patients), but at the 7-year follow-up the proportion of male patients reporting symptoms of depression was significantly higher than for female patients (9% vs. 2%; *p* = 0.018). Regarding anxiety, 10% of the female patients and 9% of the male patients reported symptoms of anxiety above the clinical cut-off at the 7-year follow-up (*p* = 0.909); this was similar to the prevalence’s seen at the post-treatment assessment (14 and 9% respectively).

### Clinical assessment of residual exhaustion/fatigue more than 7 years after seeking care

Of the 163 patients who underwent clinical assessment, 51 (31%) were judged to still be clinically exhausted due to stress-related exposure, 13 (8%) were judged to be exhausted but due to other reasons such as hypothyroidism or alcohol abuse, and the remaining 99 (61%) were judged to no longer fulfil the criteria for ED.

## Discussion

The main result of this study is that almost half of former patients treated for stress-related exhaustion reported fatigue 7 years after seeking care. Clinical assessment of a subgroup of the patients confirmed that one third were judged to be clinically exhausted due to stress exposure. The most commonly reported residual symptom was reduced stress tolerance, but many patients also reported problems with concentration and memory and sleep disturbances that plausibly are residual symptoms due to the former exhaustion.

Thus, a subgroup of patients previously seeking care for exhaustion seem to struggle with long-lasting symptoms that do not resolve with time. The treatment offered at the clinic does not seem to be the right treatment or not sufficient for these patients. Further studies are needed to elucidate if patients with long-lasting residual symptoms can be helped with other treatments or if other reasons than treatment are of central importance for this long-lasting recovery. One possibility is that some of these individuals could been suffering from other psychiatric problems that have not been dealt with, but this is unlikely since these problems most probably would have been identified during the clinical assessment. Symptoms of depression and anxiety were uncommon at follow-up, so neither depression nor anxiety can be considered as a plausible explanation for the fatigue. Another explanation could be that some of these patients are still facing severe stress exposure which has not been dealt with, resulting in continued symptoms that cannot be treated without dealing with the exposure. Some of these individuals could also be struggling with different personal traits such as overcommitment and/or perfectionism that could hinder recovery if not dealt with, particularly if these individual are still encountering poor psychosocial work environment or other high demanding stress exposure [19]. The long-lasting recovery could also be related to changes in brain function that has been seen among these patients or other biological mechanisms that plausible could be chronically affected [2]. Plausible contributors to the long-lasting recovery need to be explored in further studies.

One important reflection when considering current results is that patients included in this study were generally highly educated with high socioeconomic status, and this was the case even for the subgroup with residual fatigue symptoms. Thus, even with all plausible resources, including economic stability, social support, and high occupational attainment, recovery from exhaustion seemed to be a challenge for many of these patients.

Even though many patients do report residual symptoms, majority does confirm that they do feel better compared to when the initially sought care. However, only 16% state that they are fully recovered. None of the patients who stated that they were fully recovered reported fatigue as a remaining symptom, and this is probably the reason why they considered themselves fully recovered. Thus, perception of fatigue is clearly an important cardinal symptom, and patients who do not perceive extreme fatigue consider themselves to be fully recovered despite the presence of other symptoms such as problems with memory or sleep.

The most common residual complaint was reduced stress tolerance, reported by as many as 76% of the female patients and 64% of the male patients. One speculation is that when comparing with the situation preceding the exhaustion, the patients may feel such a difference in how much stress they can handle. Some kind of stress sensitization could also have occurred and this needs to be further explored in future studies [20].

Symptoms related to cognitive impairment are also commonly reported, particularly problems with memory and this has previously been show in several studies with shorter follow-ups [21–23]. Part of the perceived memory problems could be related to naturally occurring changes due to aging, but it is also plausible that these problems are partly related to the previous exhaustion, since nearly 60 % of the patients reported either cognitive and/or memory problems 7 years after seeking care and many of them were still relatively young.

We can only speculate that the combined perception of reduced stress tolerance, extreme fatigue, and problems with concentration and memory could affect work ability in many of these former patients as few patients are still on sick leave. Most of the participants in the present study were highly educated, and so we can assume that many of them ha cognitively demanding work situation. It is thus plausible that these residual symptoms are contributing to lost productivity at work for many of these former patients.

The pattern regarding residual symptoms seems to be similar for men and women. The only noticeable difference is that male patients report somewhat higher prevalence of depression at follow-up. Relative few men are included in this follow up but a cautious speculation is that somewhat higher rate of depression among men could be related to masculinity and denial of vulnerability, including the fact that men tend to refrain from seeking professional help for mental health problems [24].

### Strengths and limitations

To our knowledge, such a long-term follow-up of patients with clinical burnout has not been previously conducted, which is a major strength of this study. The study group consisted of over 200 patients, which is another strength. However, there are several limitations that need to be discussed. The patients were originally referred to a specialist clinic from primary care centres. Since the diagnostic criteria for ED were relatively new at the time, many primary care physicians were uncertain of the criteria and perhaps unaware of the existence of our specialist clinic. It is likely that the most severe cases were referred to our clinic, and this population might not entirely represent patients with ED in primary care. However, the study group accepting to participate did not differ from non-responders. Thus, conclusions drawn can plausible be valid for the ED group as whole that has been treated at the clinic. Studies are being conducted with the aim of comparing this specialist care unit population with patients treated solely in primary care. A large part of the measures in this study were self-reports, which is a limitation. However, several follow-up time points were used to confirm that the symptoms reported were in fact residual. One major strength of this study was the clinical assessment of a subgroup of patients used to confirm how large a proportion of the patients were in fact still exhausted. The clinical assessment could also be used to assess other diseases that might explain the residual exhaustion reported by the patients. There is currently no objective diagnostic marker available for stress-related exhaustion. We and others have previously published several interesting findings indicating that the fatigue described by these patients can partly be explained with different biological correlates, [25–28] and ongoing studies are being conducted including long-term follow-up.

### Clinical implications

Our results have several clinical implications. For many patients with exhaustion, recovery seem to take time and more knowledge that could explain such a long period of recovery is essential. Thus, neither the health care system nor the employers are prepared for such a long recovery and it is not compatible with the guidelines for sick-leave issued by the social insurance system. Early detection of ED is an urgent matter as preventive measures are needed and it has been shown that many patients with ED frequently are seeking care for different symptoms years before diagnoses with ED [29]. Even more important is to detect the subgroup of patients defined to be clinically exhausted years after seeking care. Our results confirm previous studies that this seems to be around 30% of the patient population [12, 13]. There is an urgent need to assess plausible signs that can be used for early detection of poor recovery over time. Another important clinical implication is the question of whether some of these residual symptoms are permanent, at least in a subgroup of this patient group. The perception of reduced stress tolerance could be considered as a survival mechanism, in that the tipping point for awareness of symptoms and high stress load has been changed. However, the persistent exhaustion and perception of cognitive impairments does raise the question how long-lasting the changes in brain function are that have been shown among these patients [2]. Common psychosocial stressors may result in severe long-standing physiological and psychological consequences, including increased risk of Alzheimer’s disease [29]. It is thus urgent to further elucidate these long-lasting consequences and to investigate whether some functional impairments are permanent.

Another important clinical implication related to the number of residual symptoms perceived in this patient group is the probable consequences for the patient’s work situation. The residual symptoms are most likely affecting work ability, and we have previously shown that individuals with self-rated exhaustion have a ten times higher risk of reporting poor work ability 2 years later [30]. Thus, it is of utmost importance that collaboration is established between the health care provider and the workplace, since in many cases there will be a need for adjustment to work conditions. It would be preferable for these patients to be attended by occupational health services, since knowledge of workplace adjustment is needed for successful return to work. This group of patients need to be taken seriously by the health care system, the social insurance system, the employer, and society in general.

## Conclusion

The main conclusion of this study is that a third of former patients with exhaustion disorder are still clinically considered exhausted 7 years after seeking care. In fact, most former patients with exhaustion disorder report residual symptoms such as decreased stress tolerance, extreme fatigue, sleep problems, and cognitive problems. We found no substantial differences when comparing men and women, confirming our previous results that stress-related exhaustion seems to affect both sexes in a similar manner. The long-lasting residual symptoms reported by most of these former patients are a major concern, the societal cost is large, and the results from this study raise the important issue of prevention and workplace promotion. Further studies are needed to elucidate the reasons for this long-term recovery to enable the possibility of early detection of patients at risk for long-term course of symptom.

## Data Availability

The datasets used and analysed during the current study are available from the corresponding author on reasonable request.

## Abbreviations

ED: Exhaustion disorder
HADS: Hospital Anxiety and Depression Scale
MBI: Maslach Burnout Inventory
SMBQ: Shirom-Melamed Burnout Questionnaire
